# Transgenic Tobacco Expressing the TAT-Helicokinin I-CpTI Fusion Protein Show Increased Resistance and Toxicity to *Helicoverpa armigera* (Lepidoptera: Noctuidae)

**DOI:** 10.3390/genes8010028

**Published:** 2017-01-12

**Authors:** Zhou Zhou, Yongli Li, Chunyan Yuan, Yongan Zhang, Liangjian Qu

**Affiliations:** 1 Key Laboratory of Forest Protection, State Forestry Administration, Research Institute of Forest Ecology, Environment and Protection, Chinese Academy of Forestry, Beijing 100091, China; zhouzhouhaust@163.com (Z.Z.); Zhangyab59@gmail.com (Y.Z.); 2 College of Forestry, Henan University of Science and Technology, Luoyang 471003, China; yonglili1978@163.com (Y.L.); haustforestry@yeah.net (C.Y.)

**Keywords:** helicokinin I, cowpea trypsin inhibitor (CpTI), transactivator of transcription (TAT), protein transduction domain (PTD), *Helicoverpa armigera*

## Abstract

Insect kinins were shown to have diuretic activity, inhibit weight gain, and have antifeedant activity in insects. In order to study the potential of the TAT-fusion approach to deliver diuretic peptides per os to pest insects, the HezK I peptide from *Helicoverpa zea*, as a representative of the kinin family, was selected. The fusion gene TAT-HezK I was designed and was used to transform tobacco plants. As a means to further improve the stability of TAT-HezK I, a fusion protein incorporating HezK I, transactivator of transcription (TAT), and the cowpea trypsin inhibitor (CpTI) was also designed. Finally, the toxicity of the different tobacco transgenic strains toward *Helicoverpa armigera* was compared. The results demonstrated that TAT-HezK I had high toxicity against insects via transgenic expression of the peptide in planta and intake through larval feeding. The toxicity of the fusion TAT-HezK I and CpTI was higher than the CpTI single gene in transgenic tobacco, and the fusion TAT-HezK I and CpTI further enhanced the stability and bioavailability of agents in oral administration. Our research helps in targeting new genes for improving herbivore tolerance in transgenic plant breeding.

## 1. Introduction

The insect kinins are multifunctional neuropeptides found in several arthropod and invertebrate groups [1]. Insect kinins were shown to have diuretic activity on isolated Malpighian tubules of the cricket *Acheta* [2] and the yellow fever mosquito *Aedes aegypti* [3]. Insect neuropeptides of the insect kinin class share a common C-terminal pentapeptide sequence Phe^1^-Xaa_1_^2^-Xaa_2_^3-^Trp^4^-Gly^5^-NH_2_ (Xaa_1_^2^ = His, Asn, Phe, Ser, or Tyr; Xaa_2_^3^ = Pro, Ser, or Ala). Insect kinins or their synthetic analogs have been reported to inhibit weight gain when fed to, or injected in larvae of tobacco budworm *Heliothis virescens* and corn earworm *Helicoverpa zea*, both serious agricultural pests [4,5]. Interestingly, antifeedant activity and high mortality were found in the pea aphid *Acyrthosiphon pisum* [6].

Seinsche et al. [5] demonstrated that the weight gain inhibition observed in *H. virescens* is accompanied by an increase in the excretion of water in the feces, consistent with the diuretic activity previously observed in crickets [7], flies [8,9], as well as the lepidopteran *H. virescens* [5]. The authors further speculated that the insect kinins could have induced a starvation signal in the *Heliothis* larvae, resulting in mobilization of energy stores and a decreased efficiency in exploiting digested nutrients [5]. Together with the increased excretion of fluid and the induction of a starvation response, an inhibition of digestive enzyme release may have led to the weight losses observed in both *H. virescens* [5] and *H. zea* [8] treated with insect kinins and/or analogs.

The diuretic activity of the helicokinins I, II, and III (HezK I, II, and III) from *H. zea* was tested on *H. virescens* larvae. All three kinins increased fluid secretion in isolated Malpighian tubules in a dose-dependent manner. Injections into the haemolymph caused a significant reduction in weight gain after 24 h and, in the case of HezK I, led to an increased mortality of 43% within six days, which was the most efficient of the three helicokinins [5]. No oral activity data has been reported for the insect kinin class of neuropeptides. Generally, oral activity for unmodified insect neuropeptides is poor to nonexistent, in large part due to the inability of these molecules to cross the gut epithelium.

Cell penetrating peptides (CPP) have been studied to facilitate the non-invasive (e.g., oral, transdermal) delivery of macromolecular therapeutic agents in animal models such as the mouse [10]. One of the best characterized CPP is the protein transduction domain (PTD) of the human immunodeficiency virus-1 (HIV-1) transactivator of transcription (TAT) protein. TAT-PTD is a lysine- and arginine-rich peptide of the sequence YGRKKRRQRRR that is required for TAT cell membrane transduction, and has been successfully used to transduce a variety of protein cargos into mammalian cells [11,12,13]. TAT-PTD has also been demonstrated to be useful for the systemic delivery of a galactosidase-TAT-PTD fusion protein cargo in mice via intraperitoneal injection [14], and this phenomenon could possibly be replicated in other organisms, including invertebrates. Recently, the use of TAT-PTD for the oral delivery of the *Clostera anastomosis* diapause hormone (caDH) to larvae of the moth *Helicoverpa armigera* was demonstrated [15]. The fusion peptide TAT-caDH was able to penetrate *H. armigera* midgut tissues after ingestion by third-instar larvae, and larvae exhibited pronounced growth retardation under conditions that either promoted development (27 °C, 14L:10D) or induced diapause (20 °C, 10L:14D). Under development-promoting conditions, larvae exhibited an 8% reduction in pupation rate and the duration of larval development was on average 3 days longer compared to untreated controls. Under diapause-inducing conditions, larvae fed a diet containing TAT-caDH exhibited a 14% reduction in pupation rate and the duration of larval development exhibited an increase of 12 days longer compared to controls. Fusion of the TAT peptide to a broader range of insect peptides may open up their use in pest management.

In this report, we selected the HezK I peptide from *H. zea* as a representative of the kinin family to study the potential of the TAT-fusion approach to deliver diuretic peptides per os to pest insects. We first designed the fusion gene *TAT-HezK I* and used it to transform tobacco plants. As a means to further improve the stability of the TAT-HezK I, A fusion protein incorporating HezK I, TAT, and the Cowpea Trypsin Inhibitor (CpTI) was designed. CpTI can inhibit protein degradation in the digestive tract [16]. Finally, we compared the toxicity of the different tobacco transgenic strains (*TAT-HezK I*, *TAT-HezK I-CpTI*, and controls) toward *H. armigera*. Our results may help in improving the management of insect pests of economically important crop plants, by targeting kinin-regulated physiological processes.

## 2. Materials and Methods

### 2.1. Expression Vector Construction

The *pBin438-TAT-HezK I*, *pBin438-CpTI*, and *pBin438-TAT-HezK I-CpTI* constructs were generated for plant transformation. First, the sequences for *TAT-HezK I*, *CpTI*, and *TAT-HezK I-CpTI* were obtained. The *TAT-HezK I* (GenBank: KX492908), *CpTI* (GenBank: KX492909), and *TAT-HezK I-CpTI* (GenBank: KX492910) coding sequences were synthesized by Sangon Biotech (Shanghai, China) with restriction enzyme sites added at each end *(BamH*I at the 5′ end and *Sal*I at the 3′ end). The synthetic constructs were cloned into pUC57 plasmid by a commercial service provider (Sangon). The *HezK I* was based on the helicokinin I coding sequence from *H. zea* [5]. The TAT sequence contained the codons encoding residues 47–57 (PTD: YGRKKRRQRRR) [17]. A nucleotide sequence encoding a flexible linker (FL, amino acids GGGGS) was inserted between the TAT and HezK I coding sequences [18]. The *CpTI* sequence was based on the modified cowpea trypsin inhibitor coding sequence [16]. The TAT-HezK I-CpTI fusion construct comprised the *TAT*, *HezK I*, and *CpTI* sequences, each separated by an FL linker coding sequence.

The *pUC57-TAT-HezK I*, *pUC57-CpTI*, *pUC57-TAT-HezK I-CpTI*, and pBin438 plasmids were digested using *BamH*I and *Sal*I (Sangon) at 37° C for 3 h. The digested DNA fragments were purified using a PCR product purification kit (Sangon, Shanghai, China) and ligated using T4 DNA ligase (Sangon). The ligation mixture was used to transform competent Top10 *Escherichia coli*. Recombinant *pBin438-TAT-HezK I*, *pBin438-CpTI*, and *pBin438-TAT-HezK I-CpTI* plasmids sequences were confirmed by restriction endonuclease digestion and DNA sequencing (Sangon).

**Figure 1:**
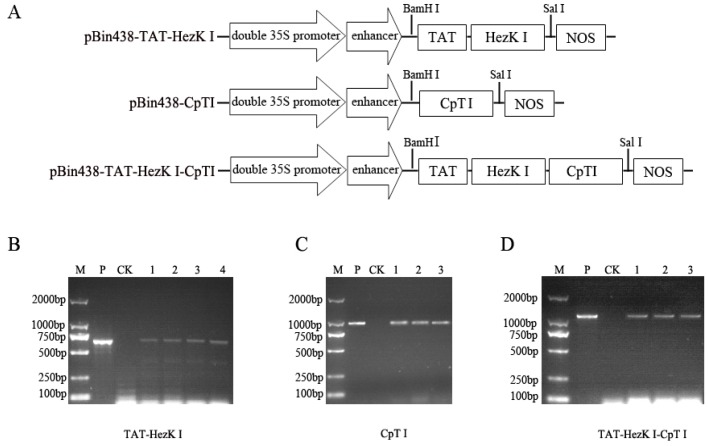
Molecular analysis of transgenic tobaccos. (**A**) Schematic representations of the *TAT-HezK I* (transactivator of transcription, TAT), *CpTI*, and *TAT-HezK I-CpTI* expression constructs, based on the pBin438 plasmid. Each fusion protein coding sequence was cloned downstream of a 2× CaMV 35S promoter and an Ω enhancer of TMV（Tobacco mosaic virus, TMV）to drive gene expression. The fusion protein sequence is also located upstream of a nopaline synthase (NOS) terminator sequence; (**B**) PCR analysis of genomic DNA from transgenic tobacco expressing TAT-HezK I. M, marker. P, Plasmid pBin438-TAT-HezK I as template. CK, DNA of wild-type plant as template. 1, 2, 3, and 4, DNA of TAT-HezK I transgenic line 1, 2, 3, and 4 as template; (**C**) PCR analysis of genomic DNA from transgenic tobacco expressing CpTI. M, marker. P, Plasmid pBin438-CpTI as template. CK, DNA of wild-type plant as template. 1, 2, and 3, DNA of CpTI transgenic line 1, 2, and 3 as template; (**D**) PCR analysis of genomic DNA from transgenic tobacco expressing TAT-HezK I-CpTI. M, Marker. P, Plasmid pBin438-TAT-HezK I-CpTI as template. CK, DNA of wild-type plant as template. 1, 2, and 3, DNA of TAT-HezK I-CpTI transgenic line 1, 2 and 3 as template.

For the purpose of overexpression, the *TAT-HezK I*, *CpTI*, and *TAT-HezK I-CpTI* genes were cloned into the plant binary expression vector pBin438, under the control of the double CaMV 35S promoters with a nopaline synthase (NOS) terminator sequence (Figure 1A). The neomycin phosphotransferase II (nptII) gene was used as a selectable marker gene. Recombinant *pBin438-TAT-HezK I*, *pBin438-CpTI*, and *pBin438-TAT-HezK I-CpTI* plasmids were then transformed into *Agrobacterium tumefaciens* stain LBA4404 by electroporation.

### 2.2. Tobacco Transformation

Gene transfer experiments were performed on the *Nicotiana tabacum* cv. K326 strain. Tobacco leave discs were transformed with the *A. tumefaciens* strain LBA4404 by suspension in MS (Murashige and Skoog) liquid medium solution [19]. Leaves of 15- to 25-day-old plantlets K326 clone at tissue culture stage were used as explants for transformation. *A. tumefaciens* strain LBA4404 harboring the *pBin438-TAT-HezK I*, *pBin438-CpTI*, or *pBin438-TAT-HezK I-CpTI* were used to transform K326 via the leaf-disc method [20]. Briefly, infected leaf discs were grown in MS basal medium supplemented with 1.0 mg/L of 6-benzyl aminopurine (6-BA) and 0.1 mg/L of naphthaleneacetic acid (NAA) in the dark for two days at 25 °C, and then transferred on the same medium with 50 mg/L kanamycin under a 16 h/8 h light/dark regime. Individual regenerated shoots were removed and induced for rooting on 1/2 MS medium supplemented with 0.1 mg/L NAA and 50 mg/L kanamycin. The plants were grown in a greenhouse at 27 °C under constant illumination.

### 2.3. PCR Analysis of Genomic DNA from Transgenic Tobacco Plants

Primary rooted transformants at the tissue culture stage were screened by genomic PCR using a 35S promoter and Nos terminator specific primer pairs. Genomic DNA of T1 generations of transgenic tobacco were isolated from transgenic plants using a Rapid Plant Genomic DNA Isolation Kit B518231-0100 (Sangon). To confirm the insertion of transgenic constructs expressing *TAT-HezK I*, *CpTI*, and *TAT-HezK I-CpTI*, PCR analysis of genomic DNA was performed with two primer pairs as follows: forward 35S promoter primer: 35S-F, 5′-GGAAACCTCCTCGGATTCCAT-3′ and backward Nos terminator primer: NOSTR, 5′-CTCATAAATAACGTCATGCATTAC-3′.

### 2.4. Target Gene Expression Analysis by Quantitative Real-Time PCR (qRT-PCR)

Total RNA of T1 generation of transgenic tobacco was extracted using the Spin Column Plant total RNA Purification Kit (Sangon) according to the manufacturer’s instructions. First-strand cDNA was generated from 4 μg RNA primed with oligo (dT)_18_ using the AMV First Strand cDNA Synthesis Kit (Sangon). qPCR analysis was performed in a 20 μL volume using ABI SybrGreen PCR Master Mix (ABI, Carlsbad, CA, USA), and the results were analyzed using the Bio-Rad iQTM5 Real-Time PCR Detection system (Bio-Rad, Hercules, CA, USA), according to the manufacturer′s instructions. The qRT-PCR reactions were initiated with a predenaturation step at 95 °C for 30 s, followed by 40 cycles of denaturation (95 °C for 20 s), annealing (59 °C for 20 s), extension (72 °C for 20 s), and a final stage of 55–95 °C to determine dissociation curves of the amplified products. *Actin* was used as the endogenous control for normalization. The value for the lowest expressing *TAT-HezK I-CpTI* transgenic line was normalised to one. More than three tobaccos were used for each transgenic line (*TAT-HezK I*, *CpTI*, or *TAT-HezK I-CpTI*) and three PCR reaction replicates were set up for each line. Primer sequences were as follows: *TAT-HezK I* or *TAT-HezK I-CpTI* genes checking primers: *TAT-HezK I*-F, 5′-ATGTACGGCCGCAAGAAGA-3′ and *TAT-HezK I*-R, 5′-ACGCCCCAGGGGCTGAAG-3′. *CpTI* or *TAT-HezK I-CpTI* genes checking primers: *CpTI*-F, 5′-TCATACCTACCTTCAGCCATCC-3′ and *CpTI*-R, 5′-AAGACTCAGAAGGTTCATCGCT-3′. Tobacco *Actin* primers: *Actin*-F, 5′-CCCCTTGTCTGTGATAACGG-3′ and *Actin*-R, 5′- AGAATACCCCTTTTGGACTGAG-3′.

### 2.5. TAT-HezK I and CpTI Polyclonal Antibody Generation and Detection of Protein in the Transgenic Plants

Polypeptides of TAT-HezK I (YGRKKRRQRRRGGGGSYFSPWGa) and middle amino acids of CpTI and (GSNHHDDSSDEPSESSEPCCDSCa) were synthesized (Genscript, Nanjing, China), and polyclonal antibodies (anti-TAT-HezK I and anti-CpTI) were generated in rabbits, as described previously [21]. Western blot analysis of the transgenic plant materials was performed. Total soluble protein (TSP) was extracted from leaves harvested from six week old plants, 3–5 nodes below the apex. Tissues were ground to a fine powder in liquid nitrogen with a chilled mortar and pestle. TSP was extracted using the One Step Plant Active Protein Extraction Kit (Sangon) according to the manufacturer’s instructions. Concentration of TSP was determined by the BCA Protein Assay Kit (Sangon) according to the manufacturer’s protocol. For the Western blot, 20 micrograms of TSP was separated by 12% sodium dodecyl sulfate-polyacrylamide gel electrophoresis (SDS-PAGE) in 1× SDS running buffer (Sangon) at 80 V for 2 h. Proteins were electrophoretically transferred to polyvinylidene fluoride (PVDF) membranes (Millipore, Bedford, MA, USA). The primary antibodies (anti-TAT-HezK I and anti-CpTI) were used at 1:5000 dilution, and the IgG-AP was used at a 1:20,000 dilution. Detection was performed using the NPT/BCIP kit (CWBIO company, Beijing, China) as described by the manufacturer.

### 2.6. Tobacco Plants Culture

To maintain transgenic and non-transgenic tobacco lines, plants were propagated by vegetative multiplication. Leaf discs were grown in MS basal medium supplemented with 1.0 mg/L 6-benzyl aminopurine (6-BA) and 0.1 mg/L naphthaleneacetic acid (NAA) at 25 °C, under 16L:8D (photophase:scotophase). Individual regenerated shoots were removed and induced for rooting on 1/2 MS medium supplemented with 0.1 mg/L NAA. After the shoots rooted, tissue culture seedlings were transplanted and grown in pre-sterilized soil at 25 ± 1 °C and 60%–80% relative humidity under a 16 h light/8 h dark photoperiod in a greenhouse.

### 2.7. Insect Rearing

*H. armigera* and diet were purchased from Baiyun Industry Co., Ltd. (Henan, China). Larvae were reared at 27 °C with a 14 h light and 10 h dark (14L:10D) cycle under 65% ± 5% relative humidity. *H. armigera* larvae were reared in groups until they reached the third-instar and were fed individually thereafter [22,23]. Similar third-instar larvae were individually transferred to a 9 cm Petri dish containing a tobacco leaf. Fresh leaves were supplied every 24 h.

### 2.8. Feeding Bioassays for H. armigera on Tobacco Leaves

To investigate whether transgenic plants displayed any differences against insect attack, feeding bioassays were conducted with detached tobacco leaves as previously described [23] with minor modifications. Leaves from two-month-old tobacco clones of transgenic lines expressing TAT-HezK I (TAT-caDH 1–4), CpTI (CpTI 1–3), or TAT-HezK I-CpTI (TAT-HezK I-CpTI 1–3), as well as age-matched wild-type controls (CK) were used. Leaves were rinsed with sterile distilled water, air dried, cut to square (5–6 cm × 5–6 cm), and were placed on a moist filter paper disc in a 90-mm-diameter Petri dish. Five hundred microlitres of sterile distilled water was provided each day to maintain relative humidity. Thirty leaves of each of the transgenic lines were taken and a third-instar larva was released on each of these leaves. The larvae were starved for 2 h prior to release to increase propensity for feeding. Three biological replicates of 30 larvae per treatment were performed and larvae were kept on fresh foliage which were replaced with leaves of the same transgenic line each day for six days in a controlled greenhouse at 27 °C with a 14L:10D photoperiod and 75% relative humidity. Larval fresh weights were recorded at d 0, d 2, d 4, and d 6 after exposure to the leaves, and mortality was recorded at d 6.

### 2.9. Statistical Analysis

All of the data were subjected to a one-way analysis of variance using the DPS software, version 7.05 (Zhejiang University, Hangzhou, China). The Student-Newman-Keuls test was used to evaluate the intergroup differences. The results of larvae weight in which “*” denotes *p* < 0.05 and “**” denotes *p* < 0.01 were considered to represent statistically significant differences to control. The data are presented as the mean ± standard error (SE).

## 3. Results

### 3.1. Generation of Transgenic Plants Expressing TAT-HezK I, CpTI, or TAT-HezK I-CpTI

The TAT-HezK I, CpTI, and TAT-HezK I-CpTI recombinant proteins were expressed via transgenic tobacco plants to assess the impact of oral delivery in *H. armigera* larvae. Transgenic tobacco plants were used in feeding assays, along with untransformed wild-type plants as controls. The *TAT-HezK I*, *CpTI*, and *pBin438-TAT-HezK I-CpTI* constructs were cloned in the pBin438 vector plasmid which drives expression with a 2× 35S promoter and an Ω enhancer of TMV (Tobacco Mosaic Virus) (Figure 1A). At least three independent transgenic T1 lines were selected for each construct. To confirm the successful insertion of each construct, genomic DNA from the transgenic T1 plants was subjected to PCR screening with specific primers of 35S-F and NOSTR (Figure 1B: TAT-HezK I 1, 2, 3, and 4; Figure 1C: CpTI 1, 2, and 3; Figure 1D: TAT-HezK I-CpTI 1, 2, and 3). PCR analysis of plant genomic DNA showed that four *pBin438-TAT-HezK* I-transformed, three *pBin438-CpTI*-transformed, and three *pBin438-TAT-HezK I-CpTI*-transformed tobacco plants were obtained.

**Figure 2:**
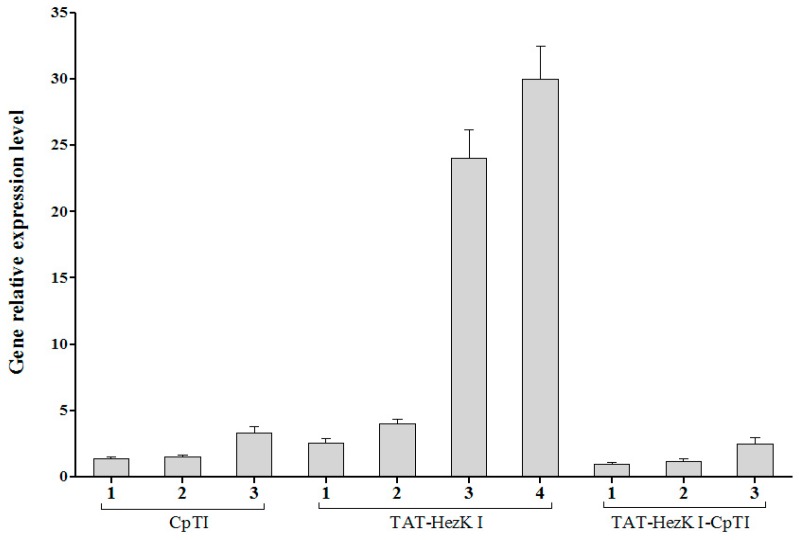
mRNA expression of CpTI, TAT-HezK I, and TAT-HezK I-CpTI in leaves of transgenic tobacco.

We used qRT-PCR to quantify the expression of each transgene. Our results indicate that the levels of *TAT-HezK I*, *CpTI*, or *TAT-HezK I-CpTI* mRNAs expressed in each of the lines were not similar to one another. Using the TAT-HezK I-CpTI 1 line as a baseline for transgene expression comparisons, we found that the TAT-HezK I-CpTI 2 and TAT-HezK I-CpTI 3 lines expressed 1.2 and 2.5 times more of the transgene, respectively. The CpTI 1, CpTI 2, and CpTI 3 lines had levels of transgene expression of 1.4, 1.5, and 3.3 times that of TAT-HezK I-CpTI 1. The TAT-HezK I 1, TAT-HezK I 2, TAT-HezK I 3, and TAT-HezK I 4 lines had levels of transgene expression of 2.5, 4.1, 24.2, and 30.3 times that of TAT-HezK I-CpTI 1, respectively (Figure 2).

### 3.2. Analysis of the Protein Expression in Transgenic Lines

**Figure 3:**
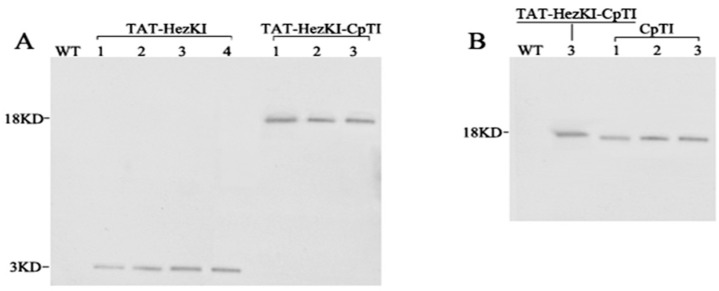
Western blotting analysis of the protein expression of TAT-HezK I, CpTI, and TAT-HezK I-CpTI in the transgenic plants. (**A**) Western blotting analysis of TAT-HezK I/TAT-HezK I-CpTI expression in transgenic lines using anti-TAT-HezK I. WT, protein extracted from WT tobacco plants. In every lane, 20 µg TSP was loaded; (**B**) Western blotting analysis of TAT-HezK I-CpTI/CpTI expression in transgenic lines using anti-CpTI. WT, Protein extracted from WT tobacco plants. In every lane, 20 µg TSP was loaded.

When probed with antiserum raised against the TAT-HezK I protein, three TAT-HezK I transgenic lines showed a major immunoreactive band of approximately 3 KDa, while three TAT-HezK I-CpTI lines showed a major immunoreactive band of approximately 18 KDa. When probed with antiserum raised against the CpTI protein, three CpTI transgenic lines expressed a single fusion protein of ca. 15 KDa, and TAT-HezK I-CpTI line 3 showed a major immunoreactive band of approximately 18 KDa. As a control, no band could be detected in the wild type (WT) plant. The evidence for the presence of immunoreactive bands corresponding in size to the predicted molecular masses for *TAT-HezK I*, *CpTI*, and *TAT-HezK I-CpTI* suggests that the introduced gene cassettes were successfully translated in planta (Figure 3). TAT-HezK I-CpTI 1–3 lines expressed similar component polypeptides. Using the TAT-HezK I-CpTI 1 line as a baseline for transgene expression comparisons, the TAT-HezK I-CpTI 2, TAT-HezK I-CpTI 3, CpTI 1, CpTI 2, CpTI 3, TAT-HezK I 1, TAT-HezK I 2, TAT-HezK I 3, and TAT-HezK I 4 lines had levels of polypeptides expression of 0.9, 1.1, 0.7, 0.9, 1.0, 0.8, 2.4, 3.6, and 3.5 times that of TAT-HezK I-CpTI 1 in the plant tissue, according to the band intensity as measured by ImageJ software 1.8.0 (National Institutes of Health, Bethesda, MD, USA).

### 3.3. Increased Resistance to, and Toxicity toward H. armigera in Transgenic Tobacco Plants Expressing CpTI, TAT-HezK I, and TAT-HezK I-CpTI

**Figure 4:**
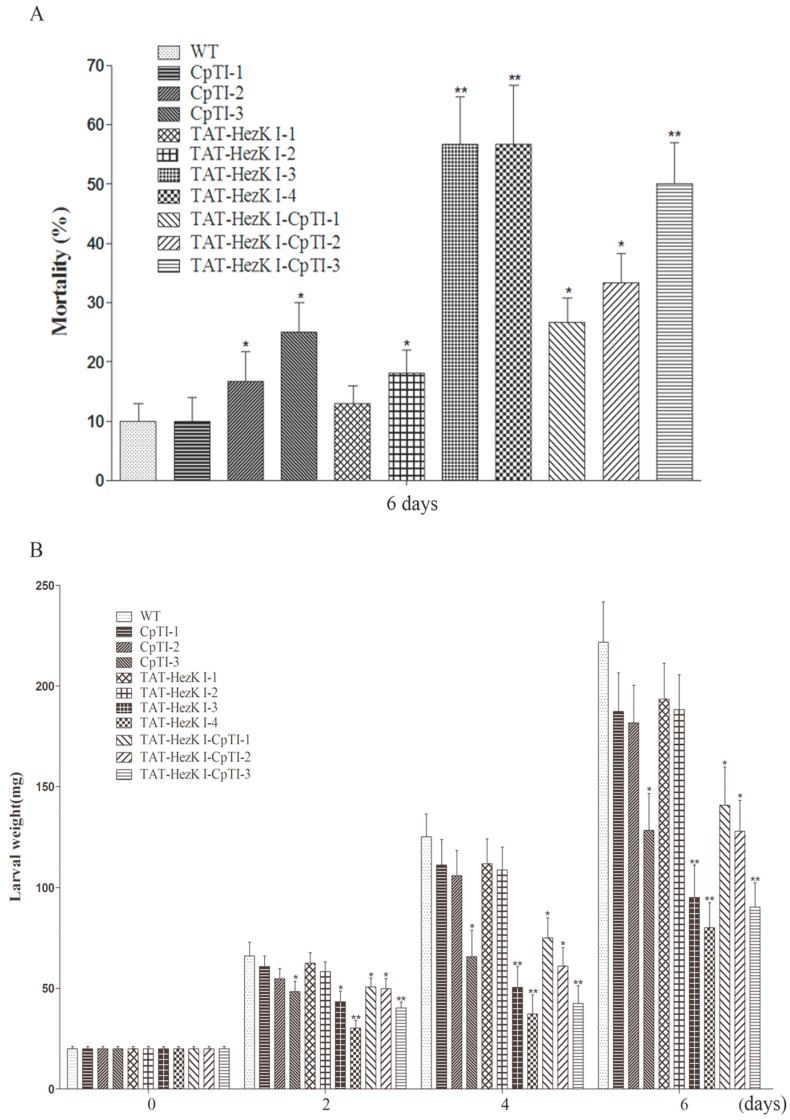
Effect of transgenic tobacco expressing CpTI, TAT-HezK I, or TAT-HezK I-CpTI proteins on larval mortality (**A**) and weight gain (**B**) as measured in feeding bioassays. Detached mature leaves of similar sizes and weights from two-month-old tobacco plants were individually placed in 9-cm Petri dishes and covered with filter paper to retain proper moisture. One third-instar were placed in each Petri dish containing a single leaf, with thirty larvae in one group. After three and six days of feeding, the larval mortality (**A**) and weight gain (**B**) were recorded. For both assays, all treatments were compared to the untransformed control (CK) (Student-Newman-Keuls test: “*” denotes *p* < 0.05 and “**” denotes *p* < 0.01).

Two-month-old tobacco plants were used for the feeding bioassays. Larvae feeding upon transgenic plants expressing CpTI, TAT-HezK I, and TAT-HezK I-CpTI exhibited a reduced survival rate. Larval mortality was assayed at d 6. Transgenic lines TAT-HezK I 3, TAT-HezK I 4, and TAT-HezK I-CpTI3 were observed with the highest levels of mortality (57%, 57%, and 50%) by feeding detached mature leaves to third-instar larvae. The larval mortality rates associated with feeding upon transgenic lines CpTI 3, TAT-HezK I 3, TAT-HezK I 4, TAT-HezK I-CpTI 1, TAT-HezK I-CpTI 2, and TAT-HezK I-CpTI 3 increased to approximately 15%, 47%, 47%, 17%, 23%, and 40% compared to the control, respectively (Figure 4A).

Larvae feeding on transgenic tobacco expressing CpTI, TAT-HezK I, and TAT-HezK I-CpTI achieved lower weight gains from d 2 to d 6 than the controls. Average larval weight was 221.7 mg when larval feeding occurred on untransformed tobacco controls. However, larval feeding on transgenic lines TAT-HezK I 3, TAT-HezK I 4, and TAT-HezK I-CpTI3 were observed with the lowest weights, and the average larval weights were 95.1 mg, 80.1 mg, and 90.3 mg at d 6, respectively (Figure 4B). When detached mature leaves were fed to *H. armigera* larvae for six days, larval average weight feeding on transgenic tobacco lines CpTI 3, TAT-HezK I 3, TAT-HezK I 4, TAT-HezK I-CpTI1, TAT-HezK I-CpTI2, and TAT-HezK I-CpTI3 were 42%, 57%, 64%, 37%, 43%, and 59% statistically less than (*p* < 0.05) feeding on untransformed tobacco controls, respectively.

## 4. Discussion

Injection of HezK I into the haemolymph of *H. virescens* larvae caused a significant reduction in weight gain after 24 h and led to an increased mortality of 43% within six days, which was the most efficient in the three described helicokinins; helicokinins I (YFSPWG-amide), II (VRFSPWG-amide), and III (KVKFSAWG-amide) [5]. For this reason, we decided to study the potential insecticidal activity of HezK I delivered in planta to *H. armigera* larvae. *H. armigera* (Lepidoptera: Noctuidae) is closely related to *H. zea*, and is a widely distributed insect pest of high agricultural importance. Here, we first designed the fusion gene *TAT-HezK I* that was transformed and expressed in tobacco used to manipulate development in agriculturally important insects.

Members of the insect kinin family are hydrolyzed and therefore inactivated by tissue-bound peptidases found in insects. Two susceptible hydrolysis sites in insect kinins have been reported. The primary site is located between the Pro^3^ and the Trp^4^ residues, with a secondary site N-terminal to the Phe^1^ residue in natural extended insect kinin sequences. Experiments demonstrate that the angiotensin converting enzyme (ACE) from the housefly can cleave at the primary hydrolysis site, whereas neprilysin (NEP) can cleave insect kinins at both the primary and secondary hydrolysis sites [4,8,24,25]. Prior to this report, no oral activity data has been reported for the insect kinin class of neuropeptides. Generally, activity for unmodified insect neuropeptides is poor to nonexistent when delivered via an oral route. The exploitation of insect feeding behavior using peptides is a valuable approach in pest control; diapause hormone (DH) were previously tested for its potential function. To reduce potential problems related to DH degradation in the insect digestive tract, the TAT-PTD peptide were used to increase the absorption of the caDH fusion protein in the insect gut. A fusion peptide that included TAT, DH, and GFP (TAT-caDH-eGFP) passed across the midgut wall and entered the hemolymph, after which it was transported to other tissues. In the previous experiments, *H. armigera* larvae feeding on a diet containing synthetic TAT-caDH were affected in their development [15]. TAT fusion partner enhanced the stability and bioavailability of the fusion protein to increase the absorption of the caDH fusion protein in the insect gut, and TAT-PTD fusion expression provides a method for the delivery of agents by oral administration. Here, our report demonstrates that TAT-HezK I can be delivered to insects via a second method, by transgenic expression of the peptide in planta and intake through larval feeding. *H. armigera* larval survival rates decreased to approximately 47% when feeding on transgenic tobacco lines of TAT-HezK I 3 and TAT-HezK I 4 at d 6. In addition, the weights of the larvae were 57% and 64% less than untransformed tobacco controls.

Previous reports indicated that the expression of the foreign CpTI protein in transgenic tobacco (*N. tabacum* L.) confers high resistance to larvae of the cotton boll-worm *H. armigera* due to the increased accumulation level of foreign CpTI protein [26]. The strategy of CpTI can offset potential problems related to protein degradation in the digestive tract and can be widely applied to other related research fields in plant genetic engineering [16]. In our research, *CpTI* transgenic tobacco were used as the positive control, and the *TAT-HezK I-CpTI* fusion gene was designed to try to further enhance the stability and bioavailability of agents in oral administration. Using suitable antibodies to detect the protein levels of CpTI and TAT-HezK I-CpTI, the expression levels of the fusion protein in transgenic lines were normalized. Though three CpTI lines and three TAT-HezK I-CpTI lines had similar target protein levels, larvae of *H. armigera* feeding on transgenic lines expressing TAT-HezK I-CpTI achieved lower weight gains; TAT-HezK I-CpTI 3 transgenic line had statistically higher mortality rates and lower weight gains on larvae than transgenic lines expressing CpTI. The *TAT-HezK I*-*CpTI* fusion gene was a better insecticidal fusion gene than the *CpTI* single gene.

TAT-HezK I 3, TAT-HezK I 4, and TAT-HezK I-CpTI 3 were the most effective lines toxic to *H. armigera* larvae. *TAT-HezK I* can be used as a kind of new gene toxic to herbivores in transgenic plant breeding. Considering mRNA expression levels, transgenic line TAT-HezK I 3 and TAT-HezK I 4 had 9.7 and 12.1 times that of TAT-HezK I-CpTI 3 in the plant tissue. However, the protein expression levels of transgenic lines TAT-HezK I 3 and TAT-HezK I 4 were 3.3 and 3.2 times that of TAT-HezK I-CpTI 3 in the plant tissue. In our opinion, the presence of the CpTI gene might help the translation of the TAT-HezK I genes when *TAT-HezK I* and *CpTI* are used. For TAT-HezK I 3, TAT-HezK I 4, and TAT-HezK I-CpTI 3, the lines had similar toxic effects to the *H. armigera* larvae; the higher TAT-HezK I protein expression level in transgenic lines TAT-HezK I 3 and TAT-HezK I 4 was the key factor cause higher toxicity. As the lower component of the TAT-HezK I-CpTI protein in the TAT-HezK I-CpTI 3 line had similar toxicity to TAT-HezK I 3 and TAT-HezK I 4 lines, the fusion TAT-HezK I and CpTI enhanced the stability and bioavailability of agents in oral administration.

## 5. Conclusions

The results demonstrated that TAT-HezK I had high toxicity against insects via transgenic expression of the peptide in planta and from intake through larval feeding. The *TAT-HezK I*-*CpTI* fusion gene was a better insecticidal fusion gene than a *CpTI* single gene. In conclusion, the studies presented here have led to the identification of interesting tools for the development of selective, environmentally friendly arthropod pest control genes capable of disrupting insect kinin regulated processes. In order to comprehensively demonstrate that the technique has value for enhancing plant resistance in field crops, bioassays will need to be repeated on intact plants in the field, and biological safety evaluations will also need to be performed in the future.
